# Diagnostic Accuracy of Magnetic Resonance Imaging in the Pre-Operative Staging of Cervical Cancer Patients Who Underwent Neoadjuvant Treatment: A Clinical–Surgical–Pathologic Comparison

**DOI:** 10.3390/cancers15072061

**Published:** 2023-03-30

**Authors:** Antonino Ditto, Umberto Leone Roberti Maggiore, Giulio Evangelisti, Giorgio Bogani, Valentina Chiappa, Fabio Martinelli, Francesco Raspagliesi

**Affiliations:** 1 Gynecologic Oncology Unit, Fondazione IRCCS Istituto Nazionale dei Tumori, 20133 Milan, Italy; 2 Academic Unit of Obstetrics and Gynaecology, IRCCS Ospedale Policlinico San Martino, 16132 Genoa, Italy; 3 Department of Neurosciences, Rehabilitation, Ophthalmology, Genetics, Maternal and Child Health (DiNOGMI), University of Genoa, 16132 Genoa, Italy

**Keywords:** MRI staging, cervical cancer, radical hysterectomy, histopathologic findings, neoadjuvant treatment, chemotherapy, radiochemotherapy, parametrium, pelvic lymph node

## Abstract

**Simple Summary:**

Magnetic resonance imaging plays a key role in preoperative staging and the evaluation of treatment responses of patients affected by cervical cancer. This is due to the ability to identify the involvement of adjacent structures such as the vagina and parametrium as well as lymph nodes. In those patients eligible for neoadjuvant treatment, the assessment of treatment response could help to plan a proper strategy to improve survival outcomes while minimizing side effects. This study aims to compare the diagnostic accuracy of magnetic resonance imaging for cervical cancer staging in subjects who underwent neoadjuvant treatment plus surgery with those who underwent direct surgery. Secondary objectives include accuracy comparison between magnetic resonance imaging and physical examination for detecting parametrium and vaginal involvement, and the accuracy in the evaluation of lymph node status. Our results show that the overall accuracy rate of magnetic resonance imaging in preoperative staging of cervical cancer does not seem to be satisfactory, especially when it is applied to pretreated patients.

**Abstract:**

Magnetic resonance imaging (MRI) has been proven to ensure high diagnostic accuracy in the identification of vaginal, parametrial, and lymph node involvement in patients affected by cervical cancer (CC), thus playing a crucial role in the preoperative staging of the disease. This study aims to compare the accuracy of MRI for the preoperative staging of patients with CC who underwent neoadjuvant treatment (NAT) or direct surgery. Retrospective data analysis of 126 patients with primary CC International Federation of Gynecology and Obstetrics stage IB3-IIB who underwent NAT before radical surgery (NAT group = 94) or received surgical treatment alone (control arm = 32) was prospectively performed. All enrolled patients were clinically assessed with both a pelvic examination and MRI before surgical treatment. Data from the clinical examination were compared with the histopathological findings to assess the accuracy of MRI for staging purposes after NAT or before direct surgery. MRI showed an overall accuracy of 46.1%, proving it to be not superior to pelvic and physical examination. The overall MRI accuracy for the evaluation of parametrial, vaginal, and lymph node status was 65.8%, 79.4%, and 79.4%, respectively. In the NAT group, the accuracy for the detection of parametrial, lymph node, and vaginal involvement was lower than the control group; however, the difference was not significant (*p* ≥ 0.05). The overall accuracy of MRI for the preoperative staging of CC after NAT is shown to be not unsatisfactory. The limits of MRI staging are especially evident when dealing with pre-treated patients.

## 1. Introduction

Cervical cancer (CC) represents the fourth most frequently diagnosed cancer and the fourth leading cause of cancer death in women globally [1,2,3]. Accurate staging of CC is crucial to plan the most appropriate treatment. Based on the International Federation of Gynecology and Obstetrics (FIGO) 2009 staging system, the clinical evaluation of CC has been exclusively based on physical and pelvic examination, a standard chest X-ray, and cystoscopy and proctoscopy if bladder and rectum invasion is suspected [4]. However, this approach could lead to an inaccurate assessment of the local extent of tumor growth, since it is influenced by the clinician’s experience and patients’ or tumor characteristics. The major difficulties in the clinical evaluation of patients with CC are related to the assessment of parametrial invasion and lymph node involvement. Several authors reported discrepancies between clinical and pathological staging with values ranging from 25% in the early stages (stage < IIA) to 65–90% in the advanced stages (stage > IIB) [5,6]. Another weakness of physical examination is that it does not allow the assessment of pelvic and lumbo-aortic lymph node involvement, which is known to deeply affect the prognosis and management of CC [7,8]. In fact, patients with CC without lymph node metastasis show a 5-year survival rate of 74%, while those with microscopic and macroscopic lymph node involvement show lower values of 58% and 39%, respectively [9].

In 2018, the revised FIGO staging system introduced new features including the use of imaging techniques as a complementary tool in the clinical diagnostic workup, also implementing the lymph node’s involvement as a separate stage (stage IIIC) [4,10,11,12]. Additionally, stage IB has been further divided into three subgroups considering the different prognoses and indications for fertility preservation surgery [13]. Since 2018, the role of imaging has been growing, although the last FIGO staging update does not provide a mandatory indication of which radiological method should be used [11].

A growing body of evidence has reported that magnetic resonance imaging (MRI) could play a relevant role in the pre-operative staging of CC [14,15,16,17]. MRI showed an accuracy of 85% in the diagnosis of parametrial involvement and 100% for vaginal infiltration [18]. Data from the literature reported that MRI has an accuracy of 85–95% for the assessment of metastatic lymph nodes [19,20,21]. Many retrospective studies have shown that the accuracy of MRI in the early stages of CC is higher than the advanced stages [22,23,24]. T2-weighted MRI provides enhanced details of the cervical anatomy, allowing the better detection of CC spreading in the surrounding structures [25]. Moreover, the functional imaging provided by diffusion-weighted imaging (DWI) and dynamic contrast-enhanced magnetic (DCE) resonance might help to highlight small neoplastic tissue as well as tumor angiogenesis [26].

MRI is the most commonly adopted tool for the assessment of the primary disease’s local extent and the evaluation of treatment response. In those patients eligible for neoadjuvant treatment (NAT), the assessment of response might facilitate the planning of a tailored management strategy to improve survival outcomes while minimizing side effects. However, the MRI assessment of CC has some limitations that might be accentuated after NAT. Indeed, post-treatment edema, necrosis, and fibrosis at the level of the tumor site and the surrounding areas could mimic residual tumors, leading to an increase in false-positive findings [27].

To date, few studies with limited series have investigated the use of MRI imaging for the assessment of response to NAT; to the best of our knowledge, none of them included a comparison between MRI and physical examination.

In this retrospective study, we aim to compare the diagnostic accuracy of MRI for CC staging in subjects who underwent NAT plus surgery with those who underwent direct surgery. Secondary objectives included the comparison of MRI and physical examination accuracy rates in detecting parametrial and vaginal involvement and even the assessment of MRI accuracy for lymph node status. This paper includes the following sections: the introduction, clinical issues in MRI evaluation of cervical cancer after neoadjuvant treatment, materials and methods, results, discussion, and conclusion.

## 2. Clinical Issues in MRI Evaluation of Cervical Cancer after Neoadjuvant Treatment

MRI is widely adopted for stadiative purposes and post-treatment monitoring in patient affected by CC [28]. Recently, the adoption of less radical treatment has made it necessary to implement radiological imaging to assess the eligibility for fertility-sparing surgery or to evaluate the response to NAT in women with locally advanced disease. However, the MRI assessment carries some limits which should be considered.

In the case of microinvasive tumors (stage IA1-2), which are commonly diagnosed after conization, conventional MRI (T1- and T2-weighted scan) is not able to delineate the lesion. In this regard, recent evidence has highlighted the role of DWI to improve the detection of early cervical lesion less than <10 mm in diameter [29]. Another concern is related to the proportion of pre-menopausal women who develop CC, which has reaches nearly 36% in Western countries [30]. In this population, cervical stromal changes influenced by hormonal changes could result in high signal intensity similar to the myometrium. Again, the implementation of DWI and DCE sequences may improve the cervical tumor–stroma contrast, allowing for a more accurate estimation of fertility-sparing treatment feasibility [31,32].

For suspected locally advanced CC, MRI helps to define the involvement of the parametrium or lymph nodes, which is fundamental for the choice of proper treatment strategy (radical surgery or definitive chemoradiotherapy). MRI evaluation of lymph node status is commonly based on size criteria, as a short axis measurement >10 mm is strongly suggestive of metastatic involvement. Additionally, other features such as morphologic features, altered intensity on T2WI, and the presence of necrotic areas could improve the accuracy. Nevertheless, MRI shows unsatisfactory specificity and sensibility for detecting lymph node metastases, probably due to the presence of microscopic foci that are below the spatial resolution of MRI. Parametrial invasion is best seen on non-fat-saturated T2-weighted images. It appears as a full-thickness interruption of the normal cervical stroma with an extension of nodular or spiculated tissue into the adjacent parametrium [12]. The preservation of a stromal border thickness >3 mm excludes parametrial involvement with a specificity of 96–99% and a negative predictive value of 94–100% [33]. However, the presence of cervical edema and/or inflammation secondary to a recent biopsy or necrosis phenomenon may be wrongly interpreted as parametrial invasion.

Potential vaginal involvement may not be evaluable in cases of a large cervical mass stretching the vaginal walls. In this case, vaginal gel administration might be a useful and cheap strategy to improve the accuracy [34].

Few studies have investigated the diagnostic performance of MRI in detecting residual pathological disease after NAT. However, these studies are of limited comparability because they are based on small case series and different methodologies. After NAT, the limitations of MRI become even more evident as edema, necrosis, and inflammation might persist for up to six months, thus making it challenging to assess the residual disease. As a result, oncologists may face a non-negligible rate of false negative/false positive cases.

In their study, Sironi et al. [35] compared MRI with surgical pathologic findings in 21 patients with CC (more than 3 cm in diameter) after NAT. The size of the tumor was correctly estimated based on MRI in 17 out of 21 cases (81.0%) and slightly overestimated in four cases (19.0%). Manfredi et al. [36] investigated the MRI accuracy in the evaluation of tumor response after concurrent chemotherapy and radiation therapy in 18 patients with locally invasive CC. Of a total of 18 patients, 4 patients (22.2%) had false-positive MRI findings. The authors reported that MRI hyperintense findings mistaken for residual disease were due to focal hyperplasia of the endocervical glands with inflammation in three patients and necrosis in one. In the same study, 33 out of 36 (91.6%) parametrial specimens and 67 out of 72 (93.1%) vaginal fornices were correctly assessed through post-treatment MRI. The involvement of three parametrial specimens and five fornices was overestimated using MRI due to edema and inflammation. In a retrospective review, Vincens et al. [27] evaluated residual disease via MRI after chemoradiation in 44 patients with stage IB2/II cervical carcinoma. The false-positive and false-negative rates were 50% and 17%, respectively. More recently, Gui et al. [37], in their retrospective study, reported a false-positive rate of 27% in post-chemoradiotherapy MRI assessment.

An explanation for the high rate of false-positive cases could be the short interval time (21–60 days) between the end of the adjuvant treatment and the radiological evaluation. Hatano et al. [38] suggested an increased accuracy of MRI when performed 3 months after the treatment in a series of patients who received external radiotherapy. Additionally, although the appropriate time interval between the end of NAT and surgery has not yet been defined, it should be considered that the effect of radiation therapy might persist during the interval between MRI and surgery.

On the other hand, the false negative rate of MRI in the evaluation of tumor response after NAT also proved to be unsatisfactory. In the study by Ferradina et al. [39], almost half of the MRI-negative cases showed the presence of a residual tumor, which was even greater than 10 mm in 22.2% of the cases. Moreover, 36 false-negative lymph nodes observed during MRI were reported in this study, of which 23 (63.9%) were shown to have a residual tumor below the threshold of imaging detection (≤10 mm). These data should be considered when planning a less radical hysterectomy, or even avoiding it, based on a negative radiologic assessment, as it could result in a risky underestimation of the residual tumor.

Recently, research has been focused on the use of DWI, which provides an enhanced assessment of residual disease as it appears as an area with a high signal, especially a high b-value, associated with lower ADC values compared to the cervical stroma. Low ADC values are related to cellular density, thus being indicative of tumor activity, while high ADC values are suggestive of edema or inflammation [40]. Two recent systematic reviews of monitoring treatment response in patients undergoing treatment for locally advanced CC supported the additional use of DWI [41,42]. Recently, Gui et al. [43], in their prospective study, reported DWI as being useful for predicting response after NAT in locally advanced CC. The authors reported the best results with the combination of T2 sequences, DW-MRI, and quantitative measurement of ADC mean values. They identified an ADC mean value ≤ 1.1 × 10^−3^ mm^2^/s as the best cutoff value to predict partial pathological response.

## 3. Materials and Methods

### 3.1. Study Design and Ethics

Data of consecutive patients with newly diagnosed CC undergoing either NAC plus radical surgery or direct surgery, treated at a tertiary referral center for gynecologic oncology in Northern Italy (Fondazione IRCCS Istituto Nazionale dei Tumori, Milan, Italy), were retrospectively reviewed between January 2011 and September 2019.

A total of 126 patients with CC of any histology underwent NAT before radical surgery (NAT group, n = 94) or received surgical treatment alone (control group, n = 32); each patient was evaluated preoperatively with physical/pelvic examination and MRI.

NAT variably consisted of carboplatin (AUC5, day1 q21) plus paclitaxel (80 mg/mq, day 1, 8, 15 q21) in a “dose dense” schedule for three cycles, the TOPOCIS regimen (topotecan 2 mg plus cisplatin 40 mg/mq) for 6 cycles, the TIP regimen (taxole 175 mg/mq plus ifofosfamide 5 gr/mq plus cisplatin 50 mg/mq) for three cycles, and the TAP regimen (taxole 135 mg/mq, adriamycin 45 mg/mq, cisplatin 50 mg/mq) for three cycles. Neoadjuvant chemoradiotherapy comprised standard pelvic radiation (external beam radiotherapy plus brachiterapy) with 5 cycles of cisplatin (40 mg/mq).

According to the FIGO staging guidelines, patients were clinically assessed with a standard physical and pelvic examination, performed under anesthesia. A parametrium with nodularity or increased thickness as well as a vagina with an exophytic lesion or increased thickness were considered positive for tumor involvement in the pelvic examination. Patients were treated with NAT before radical surgery or directly underwent surgery. The choice between direct surgery and NAT was based on the age and performance status of the patient, disease characteristics and tumor stage.

Pelvic MRI scans were acquired before the surgery, and within 4 weeks from the end of the NAT. MRI scanning was performed using a 1.5T MRI-Vision Siemens, Erlangen, Germany. A high-resolution (512 matrix) T1-T2-weighted scan was performed through the pelvis in the axial, transverse, and coronal planes (T2W TR 4325, Te 128, Fa 160°, Th 5 mm, matrix 276 × 512 Nex 3) (Tt1W Tr 670, Te 12, Fa 180, Th 5 mm, matrix 138 × 512, nex 5) before and after the injection of the contrast agent (Gd DTPA) using a 5 mm slice thickness resolution.

The radiologist who evaluated the MRI was an expert in the field of gynecologic oncology and was blinded to the FIGO clinical stage or histopathologic findings. The criteria used to investigate parametrial involvement was based on the disruption of the hypointense cervical stroma with irregular tumor signal intensity extending into the parametrium. Vaginal involvement was suspected based on the replacement of the normal low-intensity vaginal wall by a high-intensity tumor. Lymph nodes whose short diameter measured more than 1 cm were considered pathological.

Short-term treatment response after NAT was evaluated according to Response Evaluation Criteria in Solid Tumors (RECIST), Version 1.1. Complete response was defined as the disappearance of all target lesions; partial response was defined as at least a 30% decrease in the sum of diameters of target lesions, taking as a reference the baseline sum diameters; progressive disease was defined as at least a 20% increase in the sum of diameters of target lesions or the presence of new lesions; and stable disease was defined as neither sufficient shrinkage to qualify for partial response nor sufficient increase to qualify for progressive disease.

Surgery was performed within one week after the MRI scan. Patients underwent a Class II/Type B or nerve-sparing Class III/Type C1 radical abdominal hysterectomy and bilateral pelvic lymphadenectomy +/− lumbo-aortic lymphadenectomy.

The pathological assessment of the surgical specimens included the assessment of parametrial, vaginal, and lymph nodal involvement. Surgical specimens were fixed in 10% zinc-buffered formalin, routinely processed with paraffin embedding, sectioned at 5 microns of thickness, and stained with hematoxylin-eosin.

Histological classification was defined according to the World Health Organization 2014 guidelines. The T stage was determined according to The American Joint Committee on Cancer TNM classification. In the case of T1b stage, a further subclassification into pT1b1 (<2 cm), pT1b2 (2–4 cm), and pT1b3 (≥4 cm) group was performed according to the FIGO 2018 staging system.

The study was approved by the institutional review board.

### 3.2. Statistical Analysis

Findings from the pre-operative MRI were correlated with the histopathological findings to assess accuracy (A), sensitivity (Se), specificity (Sp), positive predictive value (PPV), and negative predictive value (NPV).

Descriptive statistics were used to examine associations among variables, with figures given for accuracy (A), sensitivity (Sens), specificity (Spec), positive predictive value (PPV), and negative predictive value (NPV), defined according to the following formulae:

A: TP + TN/total number of patients

Sens. = TP/(TP + FN) × 100

Spec = TN/(FP + TN) × 100

PPV = TP/(TP + FP) × 100

NPV = TN/(TN + FN) × 100

Ninety-five percent interval confidence was calculated by means of binomial distribution. Histological results were used as the standard of reference. Data analysis was performed using SPSS 8.0 software (SPSS, Chicago, IL, USA).

## 4. Results

A total of 126 patients were included in this study. Demographic characteristics of the enrolled population are reported in Table 1. The median age was 55 years (range: 35 to 74 years). In the NAT group (n = 94), 82 patients received neoadjuvant chemotherapy, and 12 received concurrent chemoradiotherapy. In the control group, a total of 32 patients underwent radical surgery. Histopathological findings reported 86 (68.3%) squamous, 32 (25.4%) adenocarcinoma, and 8 (6.3%) adenosquamous CC.

According to MRI, the overall response rate after neoadjuvant therapy was 79.2%, with 23.1% revealing a complete pathologic response.

Table 2 reports the sensitivity, specificity, accuracy, PPV, and NPV for parametrial, vaginal and lymph node status assessed with both pelvic examination and MRI.

When compared with histopathological findings, the overall accuracy of MRI in identifying parametrial, vaginal, and nodal status was 73.4% (CI 95%: 58.6–88.2).

The comparison of pelvic examination and MRI for the evaluation of parametrial and vaginal status showed an accuracy rate of 83.0% (CI 95%: 73.1–92.8) vs. 65.8% (CI 95%: 53.5–78.1), and 78.0% (CI 95%: 67.5–88.5) vs. 79.4% (CI 95%: 69.4–89.4), respectively (*p* > 0.05).

Parametrial invasion was histologically confirmed in 28 patients. The overall MRI accuracy for parametrial status was 65.8% (CI 95%: 53.5–78.1). The sensitivity and specificity of MRI for parametrial involvement in the NAT group were 49.8% (CI 95%: 35.8–63.8) and 67.3% (CI 95%: 54.2–80.4), while they were 50.1% (CI 95%: 26.2–74.0) and 82.9% (CI 95%: 66.1–100.0) for the control group, respectively. The accuracy of MRI identification of parametrial disease in the NAT group was lower than in the control group (62.9% CI 95%: 49.7–76.1 vs. 75.5% CI 95%: 65.0–86.0), although this was not statistically significant (*p* > 0.05). A total of 28 false-positive cases were identified after MRI evaluation of parametrial status. Of these, 20 (71.4%) received NACT or neoadjuvant chemoradiation.

Vaginal involvement was histologically identified in 30 patients.

The overall MRI accuracy rate for vaginal status was 79.4% (CI 95%: 69.4–89.4). The sensitivity and specificity of MRI for vaginal involvement in the NAT group were 46.9% (CI 95%: 32.0–61.8) and 91.6% (CI 95%: 83.8–99.4), respectively; in the control group, sensitivity was 56.4% (CI 95%: 43.5–69.3). The MRI accuracy for both groups in the evaluation of vaginal involvement was not significantly different (78.2% CI 95%: 66.4–90.0 vs. 81.1% CI 95%: 62.1–100; *p* < 0.05). There were 18 false-negative cases during the MRI evaluation of vaginal status. Six cases out of 18 (33.3%) had a vaginal stromal depth of invasion < 5 mm during the histopathological examination.

Lymph node metastases were histologically confirmed in 24 patients. The overall MRI accuracy rate for lymph node status was 79.4% (CI 95%: 68.1–90.7). The sensitivity and specificity of MRI for lymph node involvement in the NAT group were 29.8% (CI 95%: 17.4–42.2) and 91.4% (CI 95%: 83.9–98.9), while they were 50.1% (CI 95%: 26.4–73.8) and 84.8% (CI 95%: 62.6–100) for the control group, respectively. The MRI accuracy for both groups in the evaluation of nodal status was not significantly different (80.1% CI 95%: 69.1–91.1 vs. 80.9% CI 95%: 61.6–100; *p* ≥ 0.05). Patients with lymph node metastases belonged to the following pre-operative FIGO stages: 8 were stage IB, 2 were stage IIA, and 12 were stage IIB. One patient with a complete pathologic response after NAT had pelvic lymph node metastasis at the final pathological evaluation.

## 5. Discussion

In this study, MRI and pelvic examination showed similar accuracy in terms of the evaluation of the parametrial and vaginal status in patients with CC, regardless of NAT. Our data showed that the overall staging accuracy of MRI for parametrial, vaginal and nodal involvement was 73.4% (CI 95%: 58.6–88.2), a slightly lower value than the values reported in the literature, ranging from 77% to 86% [44,45,46]. In some cases, the radiological evaluation was challenging due to the lack of contrast between the normal cervical stroma and the tumor. The reconstitution of low-SI cervical stroma on T2WI indicates a complete response to NAT; however, increased SI from NAT-induced edema, inflammation and necrosis might persist for up to 6 months, mimicking a residual tumor [27]. Current evidence supports the potential role of diffusion-weighted imaging (DWI) in improving the detection of residual cervical tumor [43,47].

In our study, the accuracy of pelvic examination and MRI for the evaluation of parametrial involvement was 83.0% (CI 95%: 73.2–92.8) and 65.8% (CI95%: 53.5–78.1), respectively. However, this difference was not statistically significant.

Short-axis T2WI (perpendicular to the cervix) has been found to be superior to axial T2WI for the evaluation of parametrial regions [48]. Preservation of the outer-rim low-SI cervical stroma on T2WI excludes parametrial invasion with a highly negative predictive value [49]. Full-thickness cervical stromal invasion does not always signify parametrial invasion. The diagnosis of parametrial invasion requires full-thickness cervical stromal invasion and one of the following additional findings on T2WI: a speculated tumor-to-parametrial interface, a tumor nodule in the parametrium and/or tumor encasement of parametrial vessels [50].

In the evaluation of parametrial involvement, our results showed lower values for MRI in terms of specificity (70% vs. 91–99%), PPV (35% vs. 67–85%), NPV (83% vs. 95–100%) and accuracy with respect to those reported in the literature [26,44,45,51]. The overall MRI accuracy rate was 65.8% (95% CI: 53.5–78.1), significantly lower than the available literature (85–100%) [44].

In contrast, in a meta-analysis, Thomeer et al. [52] demonstrated that MRI has significantly better sensitivity (84% vs. 40%) and comparable specificity (92% vs. 93%) to pelvic examination.

The discrepancy between our study findings and the available literature about the reliability of MRI to assess parametrial status can be explained primarily by the high rate of subjects undergoing NAT compared to other studies.

In fact, the MRI evaluation of parametrial involvement in locally advanced cases might be affected by the inability to distinguish neoplastic tissue from NAT-induced phenomena; as a consequence, the rate of false-positive cases increases while specificity, PPV, and accuracy reduce. Secondly, surgical procedures after NAT included class III/type C1 radical hysterectomy, in contrast to the class II/type B radical hysterectomy commonly reported in other studies. The choice of higher surgical radicality results in an increased likelihood of false-positive cases due to the detection of parametrial involvement missed by MRI. This could explain the lower sensitivity, lower negative predictive value, and lower accuracy of MRI in the evaluation of the parametrium compared with other authors. Moreover, in previous studies, the method of histological evaluation of the parametrium, vagina or lymph nodes is not always reported accurately, and there could be a higher or lower rate of positive cases. This could also explain the difference noted in the accuracy of MRI in staging CC.

In a recent meta-analysis by Woo et al. [53], MRI showed a pooled sensitivity of 76%, a specificity of 94%, and an accuracy of 94% for detecting parametrial invasion. The use of 3T MRI, the addition of DWI to T2WI as well as the administration of antispasmodic agents were associated with superior diagnostic performance for identifying parametrial invasion [53]. Studies investigating the DWI demonstrated higher sensitivity (81% vs. 75%) and specificity (97% vs. 85%) compared to studies that did not employ DWI [54]. Another study compared two-dimensional and three-dimensional transvaginal ultrasound with MRI as the gold standard for the assessment of parametrial infiltration of CC [55]. The study results showed that the percentage agreement between 2D ultrasound and MRI (yes or no) was 76% (kappa = 0.459), while that between 3D ultrasound and MRI was 79% (kappa = 0.508). Despite their moderate similarity with MRI, 2D and 3D ultrasound share the advantage of being less costly and more readily available than MRI, thus representing a valuable tool for the preoperative work-up of CC.

The accuracy rates of MRI and pelvic examination for vaginal involvement were 79.4% (CI 95%: 69.4–89.4) and 78.0% (CI 95%: 67.5–88.5), respectively. MRI accuracy was similar to the values reported in the literature (81–100%) [44,45]. The sensitivity of MRI was 40.3% (CI 95%: 28.5–52.1), a lower value than the literature data (82–87%) [28,29]. This finding could be due to the response to NAT. In fact, three patients had vaginal involvement with a depth of invasion <5 mm, which is beyond the sensitivity of the MRI.

The lymph node status represents one of the most important prognostic factors in CC. In this study, the MRI accuracy and sensitivity rates were 79.4% (CI 95%: 68.1–90.7) and 32.6% (CI 95%: 21.0–44.2), respectively. Although unsatisfactory, these data are in line with literature evidence which shows a range of 75% to 97% for accuracy [44,45,56,57] and 36% to 82% for sensitivity [5,19,27,44,45,51]. Traditionally, lymph nodes whose short diameter is larger than 1 cm are considered metastatic during imaging assessment. However, the increased size of a lymph node might also be due to the inflammation process. Our study shows a high rate of false-negative cases probably, due to the ability of NAT to reduce the size of metastatic lymph nodes without sterilizing them completely. In this regard, some authors have suggested that the upper limit of the short axis diameter of a suspicious lymph node should be lowered to 0.5 cm [58,59].

Recently, some authors [60] reported that MRI has an accuracy of 80% in the pre-surgical evaluation of the minimum thickness of uninvolved cervical stroma; MRI measurements of the maximum depth of stromal invasion differed by ±9 mm from the pathological results in 95% of cases. Furthermore, a strong association was found between the depth of stromal invasion and the presence of involved lymph nodes, demonstrating that MRI could play a role in the choice of the best treatment option for patients affected by early CC [60].

In contrast, the subgroup analyses of patients who underwent NAT showed that MRI could play a limited role in the evaluation of CC. MRI identifies lymph node metastasis mainly according to size criteria (i.e., ≥1.0 cm in short axis), which yields a low pooled sensitivity of 56–61% and a high pooled specificity of 89–91% [61]. Morphological features, e.g., round shape, heterogeneous SI, spiculated borders and asymmetry relative to the other side, might improve sensitivity rates [61]. A high-b value DWI makes lymph nodes more noticeable because they appear as high-SI lesions in contrast with a low-SI background. Moreover, lymph node metastases show significantly lower ADC values compared to benign lymph nodes; however, variable cut-offs and the significant overlap in ADC values do not allow the use of ADC measurements in clinical practice.

Currently, FDG-PET/CT represents the most accurate imaging approach for lymph nodal staging. A meta-analysis by Ruan et al. [62] reported that FDG-PET/CT had a sensitivity of 72% and a specificity of 96% for the detection of lymph node invasion, although the detection of para-aortic node metastases was shown to be inferior due to their low prevalence and smaller size compared to pelvic node metastases.

According to the revised FIGO 2018 staging guidelines for CC, the European Society of Urogenital Radiology recently recommended both T2WI and DWI for staging purposes, the assessment of treatment response, and the evaluation of recurrence. In addition, two-dimensional T2WI is recommended over three-dimensional T2WI since the tumor margins are better delineated [63]. Pre-treatment MRI, including DWI, and FDG-PET/CT are recommended for radiotherapy planning and response monitoring. DCE remains optional, being mainly used for research purposes [63]. To date, although DWI is in an exploratory phase, it is yielding promising data in the field of CC assessment. However, there is still a need for further studies to evaluate the usefulness of DWI in the preoperative prediction of postoperative adjuvant treatment as well as the evaluation of response to NAT.

A comparison study evaluated the diagnostic performance of DWI and DCE imaging in the evaluation of tumor extent in patients with FIGO stage IB CC [64]. The diagnostic performance of DWI in the evaluation of stage IB CC was not statistically different from that of DCE. DWI could be preferable to DCE for the preoperative evaluation of stage IB cervical CC, since DCE requires more time and intravenous administration of a contrast agent that might impair renal function. A Chinese group [64] compared conventional MRI and DWI for the evaluation of CC patients submitted to definitive chemoradiation therapy, concluding that DWI could be used as a predictive and monitoring tool for treatment response to radiochemotherapy in patients with CC.

In summary, although MRI could improve the clinical assessment of CC, our data do not seem to demonstrate an improved staging accuracy in the population of patients with CC who underwent NAT [19,44].

This finding should be considered carefully, since this study has some limitations. Firstly, it is a retrospective analysis of a small population conducted in a single institution. Secondly, both radiologists were experienced in gynecologic cancer imaging; for this reason, our results may not be widely generalizable.

On the other hand, a strong point of our study is the uniformity of the diagnostic (MRI), therapeutic (NAT and surgical technique), and anatomopathological approaches.

Our dataset was collected using 1.5T MRI with conventional T1- and T2-weighted scans, reflecting the most used tool in clinical practice. This study also possesses the added value of comparing MRI with the pelvic examination, which still has clinical value, especially in low-resource countries. However, our findings highlight the ongoing need for new randomized prospective trials to evaluate the role of MRI in pre-treated patients with CC. Research should include and evaluate the most innovative MRI techniques, such as functional scans, including DCE and DWI MRI, or the use of endovaginal receiving coils or 3.0T MRI.

Among these innovative techniques, PET/MRI integrates the high-resolution multiplanar morphologic and functional information of MRI with the metabolic data of FDG-PET/CT, which can facilitate various steps in CC management, including initial staging, response monitoring, surveillance, and recurrence evaluation [65]. However, the implementation of this radiological technique in clinical practice remains limited by limited availability, high cost, and the need for specialized technical expertise.

Currently, there is growing interest in the field of radiomics, which allows the computerized extraction of data from cross-sectional images, enabling new information otherwise undetectable by the human eye [66].

Radiomics promises the discovery of noninvasive biomarkers, paving the way toward the realization of precision medicine [67]. Traditional radiomics analysis relies primarily on the computational extraction of user-defined features. Recently, rapid advances in deep learning have transformed image analysis through the automatic discovery of characteristic representations [68]. The combination of clinical and radiomics features can provide information to predict the behavior and prognosis of cervical cancer and make more accurate treatment decisions. Interestingly, Li et al. [69] found that treatment response was an independent factor for survival in those patients who receive NAT for CC, thus supporting the relevant role of imaging assessment.

## 6. Conclusions

In conclusion, no significant difference was found for tumor detection, parametrial, vaginal or lymph node assessment. The overall accuracy rate of MRI in preoperative staging of CC does not seem to be satisfactory. The limitations of MRI in diagnosing the disease spreading outside the cervix are particularly evident when applied to pretreated patients.

This study has some limitations, including its retrospective design including a limited sample of patients in a single institution. Both radiologists had experience in gynecologic cancer imaging, so our results may not be widely generalizable.

The strengths of our study include the uniformity of diagnostic (MRI), therapeutic (NAT and surgical technique) and anatomopathological approaches.

Meanwhile, prospective studies with large sample sizes and functional MRI techniques are necessary to overcome the limits of conventional MRI. The growing literature indicates the potential of radiomics in providing valuable information for managing clinically relevant endpoints; however, many unanswered questions need to be clarified before its implementation in clinical practice.

## Figures and Tables

**Table 1:** Characteristics of the patients included in the study.

	N (%) or Mean (Min–Max)
Number of patients	126 (100%)
Age (years)	55 (45–74)
FIGO Staging *	
IA	6 (4.8)
IB1-2	38 (30.2)
IB3	16 (12.7)
IIA	10 (7.9)
IIB	56 (44.4)
**Pathological Staging**	
0	24 (19.0)
IA	6 (4.8)
IB	54 (42.9)
IIA	14 (11.1)
IIB	28 (22.2)
**MRI Staging**	
0	14 (11.1)
IA	0
IB	62 (49.2)
IIA	8 (6.3)
IIB	42 (33.3)
**Histology †**	
Squamous cell carcinoma	86 (68.3)
Adenocarcinoma	32 (25.4)
Adenosquamous	8 (6.3)
**Extrauterine disease**	
Parametrial invasion	28 (22.2)
Vaginal infiltration	30 (23.8)
Lymphnode metastasis	24 (19.9)

* Pathologic T stage was assigned according to AJCC TNM classification. † Histological classification was assigned according to the World Health Organization (2014). Stage 0 includes those patients with carcinoma in situ or complete pathologic response after neoadjuvant chemotherapy or pre-operative chemoradiation.

**Table 2:** MRI and physical examination staging in pre-treated and not pre-treated patients affected by cervical cancer.

		Parametrial Status					Nodal Status					Vaginal Status				
Staging	Pts n°	Se% CI95%	Sp% CI95%	PPV% CI95%	NPV% CI95%	A% CI95%	Se% CI95%	Sp% CI95%	PPV% CI95%	NPV% CI95%	A% CI95%	Se% CI95%	Sp% CI95%	PPV% CI95%	NPV% CI95%	A% CI95%
MRI (whole series)	126	50.2 (37.6–62.8)	70.2 (58.1–82.3)	35.0 (23.2–46.8)	83.0 (74.7–91.3)	65.8 (53.5–78.1)	32.6 (21.0–44.2)	89.3 (83.4–95.2)	43.7 (32.8–54.6)	85.1 (76.7–93.5)	79.4 (68.1–90.7)	40.3 (28.5–52.1)	91.5 (84.3–98.7)	60.0 (48.8–71.2)	83.9 (74.0–93.8)	79.4 (69.4–89.4)
MRI (NAT group)	94	49.8 (35.8–63.8)	67.3 (54.2–80.4)	29.2 (15.5–42.9)	83.0 (73.9–92.1)	62.9 (49.7–76.1)	29.8 (17.4–42.2)	91.4 (83.9–98.9)	50.2 (36.6–63.8)	82.7 (71.4–94.0)	80.1 (69.1–91.1)	46.9 (32.0–61.8)	91.6 (83.8–99.4)	67.1 (53.2–81.0)	82.6 (70.3–94.9)	78.2 (66.4–90.0)
MRI (control group)	32	50.1 (26.2–74.0)	82.9 (66.1–100.0)	50.1 (26.9–73.3)	83.3 (68.0–100.0)	75.5 (65.0–86.0)	50.1 (26.4–73.8)	84.8 (62.6–100)	34.2 (10.1–58.3)	91.8 (79.8–100)	80.9 (61.6–100)	56.4 (43.5–69.3)	92.3 (65.5–100)	72.7 (56.5–88.9)	87.4 (70.1–100)	81.1 (62.1–100)
Physical examination (whole series)	126	49.8 (38.0–61.6)	92.1 (85.1–99.1)	62.8 (51.3–74.3)	85.6 (75.4–95.8)	83.0 (73.2–92.8)	NA	NA	NA	NA	NA	33.0 (21.9–44.1)	91.7 (84.9–98.5)	55.6 (43.1–68.1)	81.3 (71.9–90.7)	78.0 (67.5–88.5)

A: accuracy, MRI: magnetic resonance imaging, NPV: negative predictive value, PPV: positive predictive value, PTS: patients, Sens: sensitivity, Spec: specificity.

## Data Availability

Data available on request.
